# Closed Contour Specular Reflection Segmentation in Laparoscopic Images

**DOI:** 10.1155/2013/593183

**Published:** 2013-08-01

**Authors:** Jan Marek Marcinczak, Rolf-Rainer Grigat

**Affiliations:** Hamburg University of Technology, Schlossstraße 20, 21079 Hamburg, Germany

## Abstract

Segmentation of specular reflections is an essential step in endoscopic image analysis; it affects all further processing steps including segmentation, classification, and registration tasks. The dichromatic reflectance model, which is often used for specular reflection modeling, is made for dielectric materials and not for human tissue. Hence, most recent segmentation approaches rely on thresholding techniques. In this work, we first demonstrate the limited accuracy that can be achieved by thresholding techniques and propose a hybrid method which is based on closed contours and thresholding. The method has been evaluated on 269 specular reflections in 49 images which were taken from 27 real laparoscopic interventions. Our method improves the average sensitivity by 16% compared to the state-of-the-art thresholding methods.

## 1. Introduction

 One major concern in laparoscopic image processing is specular reflections which are present in the majority of laparoscopic interventions and affect all following processing. Specular reflections are most pronounced if the surface normal bisects the angle between the incident light and the camera. They are caused by moist tissue and appear as white glare or light-colored glare in the images. Many different approaches to segment specular reflections have been proposed in the previous decades. Most of them are based on the dichromatic reflection model [1, 2]. Let *i* be the incident angle, *e* the exitance angle, *g* the phase angle, and *λ* the wavelength. The reflectance *L*_*s*_(*λ*, *i*, *e*, *g*) and *L*_*b*_(*λ*, *i*, *e*, *g*) model the surface reflection and the body reflection. The radiance *L*(*λ*, *i*, *e*, *g*) reflected by a surface can be defined as

(1) $$\begin{array}{c} L\left(\lambda ,i,e,g\right)=L_{s}\left(\lambda ,i,e,g\right)+L_{b}\left(\lambda ,i,e,g\right). \end{array}$$

The dichromatic reflection model holds for dielectric surfaces and separates the spectral reflection from the geometric reflection [3–5]:

(2) $$\begin{array}{c} L\left(\lambda ,i,e,g\right)=m_{s}\left(i,e,g\right)c_{s}\left(\lambda \right)+m_{b}\left(i,e,g\right)c_{b}\left(\lambda \right), \end{array}$$

where *m*_*s*_(*i*, *e*, *g*) and *m*_*b*_(*i*, *e*, *g*) are geometric scaling factors and *c*_*s*_(*λ*) and *c*_*b*_(*λ*) are spectral power distributions. The body reflection (diffuse reflection) and the specular reflection form linear clusters in a color histogram [6]; fitting linear subspaces to these clusters can be used to detect specular reflections in images, and the diffuse color can be reconstructed by projection. However, in practice, surface roughness and the imaging geometry make the fitting of subspaces inaccurate [7]. Additionally, the assumption of dielectric surfaces is not fulfilled by human tissue. Nevertheless, several algorithms have been proposed that use the dichromatic model in an endoscopic environment [8, 9]. However, Vogt et al. show that simple S channel thresholding in the HSV color space achieves similar accuracy on endoscopic images [10]. Several adaptive thresholding techniques have been proposed that make use of nonlinear color transformations to separate the specular reflections from bright tissue in color space [9, 11]. Most of the specular reflection segmentation algorithms have in common that thresholding is used to segment the central part of the reflections, and the bright region surrounding the reflection is segmented in a second step. In the following, we will refer to this region where specular reflection is still strong, but body reflection increases as specular lobe. Commonly, this region is segmented by applying morphological operations to the thresholded image or using region growing [12]. As mentioned by several authors, single threshold techniques have limited accuracy [13, 14]. Bright parts of the tissue commonly intersect with weak specular reflections in color space. The approach of Oh et al. [14] is similar to our approach as they distinguish between weak and strong reflections using multiple thresholds. In our approach, specular reflections are classified as weak, intermediate, and strong reflections. We demonstrate that closed contours can be used to detect weak reflections which would be missed by thresholding techniques. The segmentation of weak, intermediate, and strong reflections is combined to obtain the final segmentation.

In here, Sections 2 and 3 discuss methods and results. First, thresholding techniques for specular reflection segmentation are detailed in Sections 2.1 and 2.2. These methods are applied in conjunction with the specular lobe segmentation described in Section 2.3.  Section 2.4 defines our hybrid approach combining thresholding techniques with closed contour segmentation. The results of our approach are compared to the classical techniques in Section 3.

## 2. Methods

 The most common techniques for specular reflection segmentation in endoscopic images are thresholding methods [9–11, 13, 14]. In the following sections, we detail the specular peak thresholding algorithm and outline a second thresholding method which we refer to as cone thresholding. In the evaluation, we compare these thresholding techniques to our hybrid approach which is explained in Section 2.4.

### 2.1. Specular Peak Thresholding

 A common assumption for specular reflections is that they are located in the brightest peak in the histogram of an image. Stehle selects the brightest peak of the luminance channel of the YUV color space [15], while Saint-Pierre et al. perform a nonlinear transformation. Let *I* : *Ω* → ℝ^3^ be an RGB image; the transformed image *I*_*t*_ : *Ω* → ℝ^3^ is defined by

(3) $$\begin{array}{c} I_{t}\left(x\right)=\left(1-S\left(x\right)\right)I\left(x\right), \end{array}$$

where *S*(**x**) is the saturation channel of *I*(**x**) using the HSV color space [16]. This transformation decreases the color values depending on their distance to the gray-axis. The transformation is based on the assumption that most specular reflections will be located close to the gray-axis and have a low saturation. This transformation increases the gap between specular reflections and tissue in the histogram [11]. The threshold is selected by the following criterion. Let *h*(*t*) be the histogram of the Y channel of *I*_*t*_ and *t* = {0,…, 255}. The threshold *t*_spec_ is given by

(4) $$\begin{array}{c} t_{\text{spec}}=\max\left\{t\;|\;\tilde{h}\left(t\right)-\tilde{h}\left(t+1\right)>0\right\}, \end{array}$$

with

(5) $$\begin{array}{c} \tilde{h}\left(t\right)=\{\begin{array}{cc} 1 & \text{if}\;\;h\left(t\right)-h\left(t+1\right)>0,\\ 0 & \text{if}\;\;h\left(t\right)-h\left(t+1\right)\leq 0. \end{array} \end{array}$$

In [11], the threshold *t*_spec_ is directly used to detect specular reflections. However, in our experiments this segmentation was not robust; if no specular reflections are present in the image, the brightest parts of the tissue will be classified as specular reflections. Furthermore, different specular reflections might appear at different intensity levels and lead to several peaks in the high intensity range of the histogram *h*(*t*_*i*_). Therefore, we apply the two following steps to increase the robustness of the algorithm. First, we allow only thresholds *t*_spec_ > *t*_min⁡_. Second, we convolve *h*(*t*_*i*_) with a Gaussian kernel *𝒩*(*t*_*i*_, *σ*) to merge the peaks of *h*(*t*_*i*_) that are caused by different specular reflections. The set *𝒮*⊆*Ω* of specular reflections is given by *𝒮* = {**x** ∈ *Ω* | *I*_gray_(**x**) > *t*_spec_}, where *I*_gray_ is the Y channel of *I*_*t*_. This approach sets the threshold according to the brightest specular peak in *h*(*t*_*i*_)⋆*𝒩*(*t*_*i*_, *σ*).

### 2.2. Cone Thresholding

 One drawback of the specular peak thresholding technique is the assumption that specular reflections are represented by a single peak in the histogram of an image. In practice, this is not always the case. Specular reflections appear at different intensity levels and can lead to several peaks in the histogram. Therefore, another technique which relies on defining a cone in the RGB color space as specular reflections can be used. The cone is located on the axis $n_{r=g=b}={\left(\begin{array}{ccc} 1 & 1 & 1 \end{array}\right)}^{\text{T}}$ for which *r* = *g* = *b*, which implicitly assumes a perfect white balance. Let **I** : *Ω* → ℝ^3^ with *Ω*⊆ℝ^2^ be an RGB image. The projection of **I**(**x**) on **n**_*r*=*g*=*b*_ is given by

(6) $$\begin{array}{c} p_{r=g=b}\left(x\right)=\frac{\langle n_{r=g=b},I\left(x\right)\rangle }{\left|n_{r=g=b}\right|}n_{r=g=b}. \end{array}$$

The set *𝒮*_spec_ of specular reflections is then defined by

(7) 𝒮spec={x∈Ω ∣ |I(x)−pr=g=b(x)| <a(〈nr=g=b,I(x)〉|nr=g=b|−x0)}.

The parameters *x*_0_ and *a* define the tip and the slope of the cone. This detection algorithm relies on the assumption that the specular reflections are close to the gray-axis of the RGB space. The advantage of this segmentation algorithm is that it does not suffer from multiple peaks in the histogram of the image. However, one should keep in mind that the RGB color space is a hardware-dependent color space. Therefore, the parameters *x*_0_ and *a* need to be adjusted for different hardware.

### 2.3. Specular Lobe Segmentation

 The thresholding techniques outlined in the previous sections segment the central part of the specular reflections. To obtain a segmentation of the entire specular reflection, specular lobe segmentation needs to be applied. We use a technique similar to [11] which is based on region growing [12]. Instead of using a single threshold for the region growing, we compute thresholds based on the specular reflection intensities. For every connected component *𝒮*_cc,*i*_ ⊂ *𝒮*_spec_, with *i* = 1,…, *N*, where *N* is the number of connected components in *𝒮*_spec_, the mean value of the connected component is estimated by

(8) $$\begin{array}{c} \mu_{\text{cc},i}=\frac{1}{\left|{𝒮}_{\text{cc},i}\right|}\overset{\left|{𝒮}_{\text{cc},i}\right|}{\underset{j=1}{\sum }}I\left(x_{j}\right),\;\text{where}\;\;x_{j}\in {𝒮}_{\text{cc},i}. \end{array}$$

The region growing algorithm adds a pixel **x** to the set *𝒮*_lobe_ if

(9) $$\begin{array}{c} I\left(x\right)>c\mu_{\text{cc},i}, \end{array}$$

where *c* is a scaling factor and *I*(**x**) is the luminance channel of the YUV color image. The final segmentation is given by *𝒮*_spec_ ∪ *𝒮*_lobe_. The optimal parameter *c* for different thresholding algorithms is given in Figure 3. In the following evaluation, specular lobe segmentation is used together with the outlined thresholding algorithms.

### 2.4. Hybrid Closed Contour Thresholding

**Figure 1:**
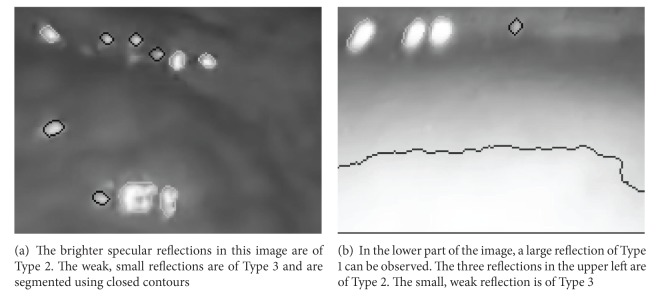
Two regions of interest show reflections of Types 1, 2, and 3. Both images are taken from laparoscopic sequences of the liver. Segmentation is performed using the hybrid contour thresholding algorithm.

**Figure 2:**
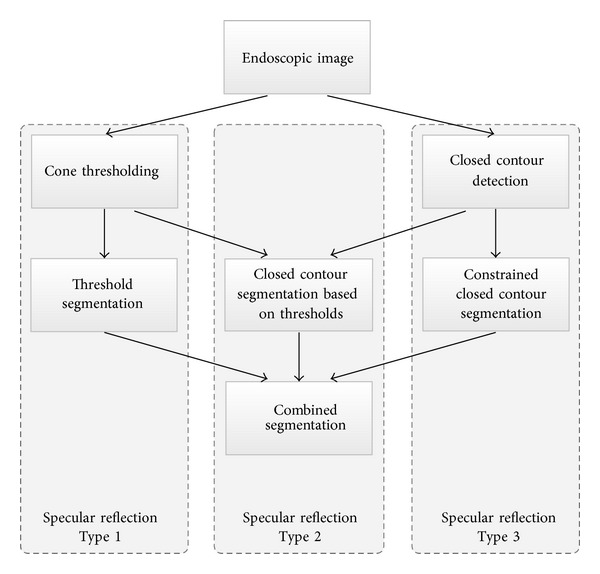
Overview of the hybrid closed contour thresholding algorithm. Three different process lines are used for the detection and are combined in a final segmentation. The contour-based segmentation is capable of segmenting even tiny specular reflections which cannot be detected by a single threshold. Furthermore, the closed contour supports reflections of Type 2, which have been detected by thresholding with a precise boundary. Reflections of Type 1 are large specular reflections with a smooth gradient and a bright central part.

 Single threshold segmentation techniques have an upper limit of accuracy which is often caused by bright tissue which is classified as specular reflection. An increase in precision can be achieved by using different models for specular reflections. In laparoscopic videos of the liver surface, different types of specular reflections appear. The first types of reflections are large specular reflections which occur in situations where the endoscope is located very close to the organ surface (Type 1). As the image intensities at specular reflections of Type 1 are very high and often clipped in the center part, thresholding can be used to segment this type of specular reflections. Another property of this type of reflection is the slowly, radial decreasing intensity. More difficult to detect are small, weak reflections located further apart from the endoscope. These reflections are caused by moist curved organ surfaces. The intensities of these small specular reflections can be low, depending on the surface geometry and the underlying tissue. However, most of this type of reflections have a step-shaped border, which can be used for detection and precise segmentation. In the proposed segmentation algorithm, this type of specular reflections is split up into small reflections with high intensity (Type 2) and specular reflections that have low intensities even in the center (Type 3). All three types are illustrated in Figure 1. For Types 2 and 3, the contour is used to determine the segmentation boundary. For every connected component in the binary image that was created by cone thresholding, it is checked if the component is enclosed by a contour; this contour is used for segmentation. If no closed contour is found, the reflection is classified as Type 1. To detect reflections of Type 3, closed contours are used as seed points. An overview of this approach is given in Figure 2. In the following, *𝒮*_Type1_ denotes the set of pixels segmented as specular reflections by thresholding. The Canny edge detector is used to compute a binary edge map *I*_Edge_(**x**) of the laparoscopic image *I*(**x**) [17]. The liver surface is smooth and lacks edges or corners. Therefore, most of the strong filter responses are caused by the boundaries of specular reflections. A morphological closing operation is used to close small gaps in the contours. Let *𝒮*_cc,*i*_ denote the set of pixels of the connected component *i* that is enclosed by the contours of *I*_Edge_(**x**). The set of Type 2 reflections *𝒮*_Type2_ is obtained by the connected components that contain at least one pixel of *𝒮*_Type1_. The weak Type 3 reflections are segmented using constraints on the connected components *𝒮*_cc_ that are not elements of *𝒮*_Type2_. The specular reflections *𝒮*_Type3_ are given by the connected components *𝒮*_cc,*i*_ that fulfill the following constraints:

(10) $$\begin{array}{c} E\left[I\left(x\right)\right]>t_{\text{av}},\;\text{where}\;\;x\in {𝒮}_{\text{cc},i},\\ E\left[I\left(x\right)\right]-E\left[I\left(\hat{x}\right)\right]>t_{\text{diff}},\;\text{where}\;\;x\in {𝒮}_{\text{cc},i},\;\;\hat{x}\in \delta {𝒮}_{\text{cc},i},\\ |{𝒮}_{\text{cc},i}|>t_{\text{cc},\min},\\ |{𝒮}_{\text{cc},i}|<t_{\text{cc},\max}. \end{array}$$

The perimeter of *𝒮*_cc,*i*_ is denoted by *δ𝒮*_cc,*i*_, and *E*[*I*(**x**)] denotes the expectation value. The last two constraints assure that only closed contours of a specific size are considered to be specular reflections of Type 3. The first two constraints are based on the aspect that the average intensity of specular reflections is limited by a lower boundary and that there should be a high decrease in intensity close to the perimeter of the reflections. The final segmentation of specular reflections is then given by *𝒮*_Type1_ ∪ *𝒮*_Type2_ ∪ *𝒮*_Type3_.

## 3. Results

**Figure 3:**
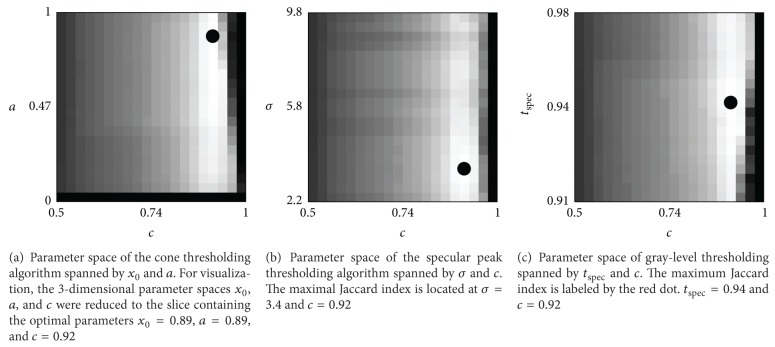
Parameter optimization using the Jaccard index. The dot labels the optimal parameter values. The optimal parameters are used in the evaluation.

 In this section, we evaluate the specular reflection algorithms outlined in the previous sections. The evaluation is performed on a dataset of 49 laparoscopic images taken from 27 patients. The images contain 269 true specular reflections. Ground truth segmentation of specular reflection is given by manual segmentation of the specular reflections. The quality of the resulting segmentation is determined using the Jaccard index [18]:

(11) $$\begin{array}{c} F_{\text{Jacard}}=\frac{t_{p}}{t_{p}+f_{p}+f_{n}}, \end{array}$$

where *t*_*p*_, *f*_*p*_, and *f*_*n*_ are the true positives, false positives, and false negatives. The Jaccard index is used to measure the overlap of a given segmentation with the ground truth segmentation. The advantage of this error metric is that the amount of true negatives is not considered; in laparoscopic images, the area of specular reflections is usually very small. Therefore, the specificity might be high although the segmented area is several times larger than the true specular reflection. The parameters for the thresholding technique were determined by maximizing the Jaccard index. Figure 3 shows the results of the parameter optimization. One of the main advantages of the hybrid segmentation is that it detects small reflections which would be missed by thresholding techniques. As the Jaccard index computes the overlap of the segmentation with the ground truth, small reflections will give only slight improvements. To demonstrate that many small reflections are detected which would be missed by thresholding, we state the sensitivity in terms of specular reflections. As the number of true negative reflections is unknown—we only have background and specular reflections—the specificity cannot be computed. Therefore, we use the positive prediction value (PPV) to consider the false positives:

(12) $$\begin{array}{c} \text{PPV}=\frac{t_{p}}{t_{p}+f_{p}}, \end{array}$$

where *t*_*p*_ and *f*_*p*_ are the number of true and false reflections detected by the segmentation algorithms. Furthermore, we use the the average Jaccard index ${\overset{-}{F}}_{\text{Jacard}}$ and the quartiles *Q*_1_, *Q*_3_ of the distribution of the Jaccard index to determine the robustness of the segmentation algorithms. The results are given in Table 1. The results demonstrate the limited accuracy that can be achieved by thresholding techniques for specular reflection segmentation; the cause for this upper limit is an overlap in the color space between specular reflections and the brightest part of the tissue. However, even the adaptive thresholding technique outlined in Section 2.1 just slightly improves accuracy. One reason for that is the assumption that the specular reflections are located in a single peak of the histogram. This assumption is not fulfilled if reflections of Type 1 and Type 3 are present in one image.

The hybrid approach using closed contours and thresholding achieves the highest sensitivity and the best segmentation results in terms of the Jaccard index. The sensitivity is increased by 16% and *Q*_1_ by 0.17 compared to the best thresholding results. Small weak reflections are detected by their closed contours and give rise to this improvement. Furthermore, the closed contours increase the accuracy of the segmentation for specular reflections of Type 2 which explains the higher Jaccard index. However, there are some closed contours that are falsely classified as specular reflections and cause the PPV to drop slightly compared to the specular peak thresholding and the cone thresholding algorithm. 

## 4. Conclusion

 In this section, it was shown that using a hybrid approach combining closed contours and thresholding significantly improves the segmentation of specular reflection in laparoscopic videos. The closed contour computation is performed using the Canny edge detector and the morphological closing operation. This process has the disadvantage that not all the contours of specular reflections will be closed and might be missed. Using approaches that always produce closed contours such as polar transformations and shortest path computations [19, 20] could increase the sensitivity. However, many open contours are not caused by specular reflections; in a brief test, closing open contours using polar transformation increased the false positives in a large scale. Therefore, we apply morphological operations to obtain all contours that contain only small gaps. This leads to a tradeoff between a high sensitivity and an adequate PPV. 

## Figures and Tables

**Table 1:** The hybrid closed contour algorithm achieves the best results compared to thresholding techniques in terms of the Jaccard index and sensitivity. Note the high value for *Q*_1_ and the sensitivity; this increase is caused by the reflections of Type 3 (T3) which are missed by the other algorithms.

Algorithm	${\overset{-}{F}}_{\text{Jaccard}}$	*Q*_1_	*Q*_3_	Sensitivity				PPV
				T1	T2	T3	Avg.	
Gray-level thresholding	0.55	0.26	0.80	1	0.96	0.10	0.74	0.96
Specular peak thresholding	0.60	0.38	**0.87**	1	0.81	0	0.61	**0.99**
Cone thresholding	0.60	0.37	0.81	1	0.91	0.10	0.70	**0.99**
Hybrid closed contour	**0.66**	**0.55**	0.82	1	**0.97**	**0.69**	**0.90**	0.96

The bold font highlights the best result for each category.
